# When ‘Best’ Practice Isn’t…

**DOI:** 10.1007/s40670-024-02048-2

**Published:** 2024-05-18

**Authors:** Gabrielle M. Finn, Megan E. L. Brown

**Affiliations:** 1 Division of Medical Education, Faculty of Biology, Medicine and Health, School of Medical Sciences, The University of Manchester, 1st Floor, Core Technology Facility, 46 Grafton Street, Manchester, M13 9NT UK; 2 School of Medical Sciences, University of Newcastle, Newcastle, UK

**Keywords:** Medical education, best practice, pedagogy, research, evidence

## Abstract

Best, is to be ‘*of the highest quality, or being the most suitable, pleasing, or effective type of thing or person*’. Within medical education, 'best-ness' is evident within best practice guides and recommendations, and within research, where best evidence influences design and conduct. Yet, much of the evidence of best-ness fails to consider best for who and where, what, and when. Thinking needs reframing, given that “best-ness” and medical education are such good bedfellows, but it is critical that we recognise the impact and influence of context — that practice can be good, but cannot be universally and unflinchingly best.

‘Best practice in medical education’ returns 4.5 million results in Google Scholar within a paltry 0.11 s. Though this result is gargantuan, it is hardly surprising, as the term ‘best practice’ is interwoven with the very fabric of our field. Throughout educational systems worldwide, from an early age, participants are encouraged to become ‘the best’ — competition is encouraged. But, what does it mean to be “the best”?

To be the best, is to be ‘*of the highest quality, or being the most suitable, pleasing, or effective type of thing or person’ *[1]. Within medical education, the concept of “best-ness” is evident within teaching, where best practice guides and recommendations are commonplace, and within research, where best evidence influences research design and conduct. Further, within curricula and assessment, best practice is applied as a benchmark for quality, a tick-box adopted regardless of contextual dependencies and differences.

Best practice, a term born within business, is a “*way of running a business or providing a service that is recognised as correct or most effective*” [2, 3]. And herein lies the problem. Within medical education, the service we are concerned with is graduating safe, competent physicians with adaptable skill sets suited to caring for their local communities. There is no one “correct” or “effective” way to achieve this aim. What’s best for a medical school, for its students, and for the communities those schools serve depends on not only global, but also local needs, and contemporary shifts in thinking. In regard to best practice, we ask: best for who, what, where, and when?

## Who, and Where?

To illuminate the importance of “who” and “where” in regard to best practice, we can take decolonisation as an example. There has been a recent surge in literature relating to decolonisation of curricula. Best practice guidance in this sphere is bound in Western perspectives, geography, and historical assumptions that mainly pertain to decolonisation in relation to the British Empire, and often ignore the influence of Nazi war crime on medical knowledge. For example, decolonisation is often presented in the context of a Black versus White racial dichotomy with ‘the absence of brown in between’ [4], essentially an absence of acknowledgment of racial diversity and local populations. The who and the where within best practice literature and discourse are Western institutions. How applicable are such practices in settings outside of the bubble of the global West? Further, best practice guidance fails to define the who and where of curricula context such as the nature of professional programme, or the where on postgraduate-undergraduate curricula continuum such guidance falls. Do such guidelines peddle notions of hierarchy that ignore context or consider all contexts homogenous — best for who and best for where? Take for example a recent paper which offers three best practice frameworks for inter-professional education [5]. One has to be best by its very definition.

## What?

“What” is another important consideration when it comes to discussing best practice. The “what” is the service or practice described itself. Often, more clarity is necessary regarding just what an innovation or practice involves, but specifically regarding the institutional context. This context might include considerations of local resources, buy in, and ‘fit’ between a practice and the way in which local healthcare is organised and structured. Longitudinal integrated clerkships (LICs), an increasingly popular model of clinical education, provide a useful example here. LICs were created in response to particular healthcare pressures and needs within the USA and Australia [6]. Though they have been applied within the UK since, recent literature suggests they may not account for the unique context of the UK healthcare system, and that the term is being used to apply to a variety of models which differ to the original, international definition [7]. The “what” of best practice here — LICs — might look different within a UK healthcare system (and within UK institutions), and a relative lack of clarity within the international literature regarding which are the essential components of the model [8] (just what the best practice involves) has led to uncertainty regarding whether all programmes utilising this approach are able to achieve the impact discussed within published research.

## When?

‘Best’ is time limited. Momentarily, let’s consider the verb ‘practise’. The more an activity is performed, the more likely improvement in performance is achieved. If practise leads to improvement, then we can always do better, and the pinnacle of ‘best’ is never quite reached. Practice is best, until it isn’t.

Claims of “best-ness” are grounded in positivist assumptions of certainty — that there is one right way that is fixed and unyielding — they neglect to consider shifts in thinking and the creation of new evidence. Take, for example, the Consolidated criteria for reporting qualitative research (COREQ) checklist [9], completion of which is lauded as best practice by some journals in the field of health professions education. Such checklists are reductionist, limited by paradigm or methodology, and most often devoid of evidence. COREQ has been problematised by experts in the field for perpetuating retired terminology or practise, or ignoring the nuances of qualitative research [10]. Yet, its use perpetuates whether explicitly labelled best-practice or not. Data saturation, which is no longer a universally recommended practice within medical education, is one such example from qualitative research where the best practice literature does not align to practise in the field [11].

## Conclusion

Given that the use of the term “best practice” can eradicate critical ‘who, what, when, and where’ provisos, we argue that “best practice” is a misnomer. It is time to extricate it from the lexicon of medical educators. But why stop there? The issue is perhaps one of global importance in the scientific world — after all bestness and the hierarchy of evidence is science-wide issues. Changing rhetoric would require a major culture shift. In a critical think piece for Forbes, businessman Mike Myatt denounces the term on the basis that individuals or organisations “*use the phrase in an authoritarian manner as a justification for the position they happen to be evangelizing*” [12]. We are scientists, not soapbox preachers. Rigorous academic and educational exploration demands attention to context that the concept of “best-ness” derides. Yes, there is extraordinary value in the sharing of practice — of ideas which worked, and which didn’t, of our experiences as educators and researchers — but we must be cautious not to label one approach as better than the rest. Guidelines should be just that — guides — not tramlines that we are bound to and unable to veer from [13]. Further, the literature supports that “best practice” is a misleading term unless measurable criteria have been systematically applied [14]. Literature recommends that adopting best practice should be exercised with caution, considering where context, processes, and values align [15]. Perhaps the alternative terms “good practice” or “evidence-based practice” would allow us to progress discourse in the field regarding our experiences as practitioners in a positive and more inclusive manner. It may take time to reframe our thinking, given that “best-ness” and medical education are such good bedfellows, but it is critical that we recognise the impact and influence of context — that practice can be good, but cannot be universally and unflinchingly best. As Mike Myatt puts it — “Best practices — aren’t”. Perhaps, best is over-rated [12]. 
